# Crystal structure of Ag_2_(μ-SCN)_2_(NH_3_)_4_

**DOI:** 10.1107/S2056989016008823

**Published:** 2016-06-03

**Authors:** Thomas G. Müller, Florian Kraus

**Affiliations:** a Anorganische Chemie, Fluorchemie, Fachbereich Chemie, Philipps-Universität Marburg, Hans-Meerwein-Strasse 4, 35032 Marburg, Germany

**Keywords:** crystal structure, silver thio­cyanate, ammonia, argentophilicity

## Abstract

Di-μ-thio­cyanato-bis­[diamminesilver(I)], [Ag_2_(μ-SCN)_2_(NH_3_)_4_], was synthesized by the reaction of AgSCN with anhydrous liquid ammonia. In the binuclear mol­ecule, the Ag^I^ atom is coordinated by two ammine ligands and the S atom of one thio­cyanate ligand. Two of these [Ag(SCN)(NH_3_)_2_] units are bridged by the S atoms of the thio­cyanate anions at longer distances, leading to a dimer with point group symmetry *C*_2_. The distance between the Ag^I^ atoms in the dimer is at 3.0927 (6) Å within the range of argentophilic inter­actions. The crystal structure displays N—H⋯N and N—H⋯S hydrogen-bonding inter­actions that build up a three-dimensional network.

## Abstract 2

The mol­ecular structure of Ag_2_(SCN)_2_(NH_3_)_4_ consists of [Ag(SCN)(NH_3_)_2_]_2_ dimers with an Ag⋯Ag separation of 3.0927 (6) Å.

## Chemical context   

The reactions of various silver salts with liquid ammonia and their products are in almost all cases still unknown. In textbooks, the formation of the linear diamminesilver(I) cation is often predicted without any structural evidence. In this contribution we want to report on the reaction and the product of AgSCN with liquid ammonia at 237 K. A dinuclear Ag^I^ complex was obtained.
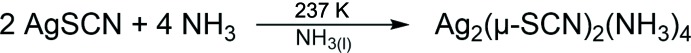


## Structural commentary   

**Figure 1:**
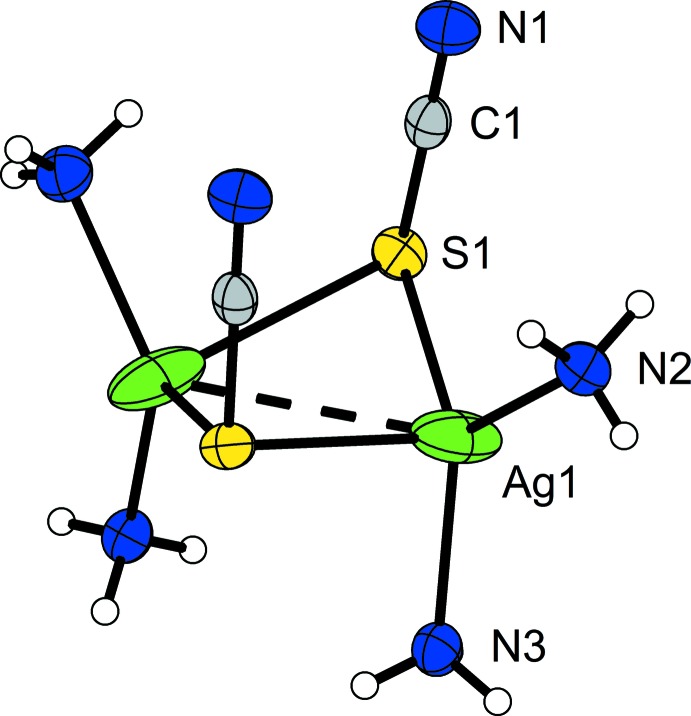
The dimeric [Ag(SCN)(NH_3_)_2_]_2_ unit in the title compound. Displacement ellipsoids are shown at the 70% probability level and H atoms are drawn with an arbitrary radius. All non-labelled atoms are generated by symmetry code (−*x*, *y*, −*z* + 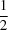).

All atoms are located on general sites. The silver atom Ag1 is surrounded by two ammine ligands (N2 and N3) with distances of 2.269 (2) and 2.248 (2) Å, respectively. These values are in good agreement with other reported Ag—N distances (Zachwieja & Jacobs, 1989 ▸). The thio­cyanate anion coordinates with its soft sulfur atom to the silver atom at a distance of 2.5363 (6) Å, which is similar compared to those of pure AgSCN (Lindqvist, 1957 ▸). The S—C—N angle in this pseudo-halide anion is with 178.2 (2)° almost linear. Two of the [Ag(SCN)(NH_3_)_2_] units are connected to each other *via* bridging S atoms of the thio­cyanato ligands into a dimer located about a twofold rotation axis (Fig. 1 ▸). The resulting coordination polyhedron around Ag1 is that of a distorted tetra­hedron where one short Ag—S distance [2.5363 (6) Å] and a long one [3.0533 (7) Å] are observed. Therefore, the bond towards the latter may be regarded as weaker. In the dimer, the two tetra­hedra are connected through one edge into a double tetra­hedron. It is inter­esting to note that the two SCN^−^ anions point in the same direction as there is no center of inversion within the mol­ecule but only the twofold rotation axis of the space-group type. The Ag⋯Ag distance is short at 3.0927 (6) Å, and is clearly in the range of argentophilic inter­actions (Jansen, 1987 ▸; Zachwieja & Jacobs, 1989 ▸; Schmidbaur & Schier, 2015 ▸).

## Supra­molecular features   

**Figure 2:**
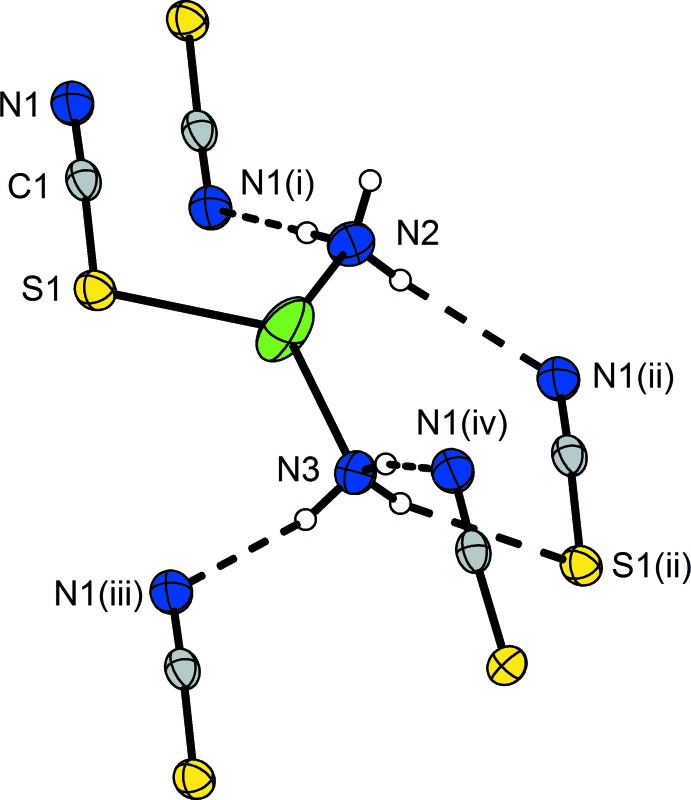
The hydrogen bonds (dashed lines) present in the structure of the title compound as illustrated for one [Ag(SCN)(NH_3_)_2_] unit with the acceptor groups of four surrounding mol­ecules. Displacement ellipsoids as in Fig. 1 ▸. [Symmetry codes: (i) −*x*, −*y* + 1, −*z*; (ii) *x* + 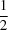, *y* − 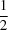, *z*; (iii) *x*, *y* − 1, *z*; (iv) −*x*, *y* − 1, –*z* + 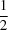.]

**Figure 3:**
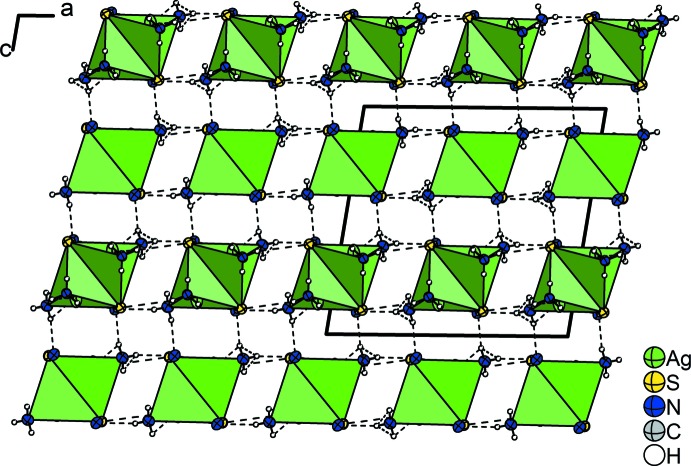
The crystal structure of Ag_2_(SCN)_2_(NH_3_)_4_ viewed along [010] with hydrogen bonds (dashed lines). Displacement ellipsoids as in Fig.1.

The dinuclear complexes are connected to others *via* hydrogen bonds between the ammine ligands (N2 and N3) as donors and the N1 and S1 atoms of the thio­cyanato ligand as acceptors. A three-dimensional network is formed in which each [Ag(SCN)(NH_3_)_2_] unit is coordinated by four (Fig. 2 ▸) and the dimer by eight other mol­ecules. Six are arranged like a hexa­gon around the central mol­ecule with all SCN ligands pointing in the same direction. Two mol­ecules reside above and below this fictitious plane and are shifted towards a corner of the hexa­gon whereby the SCN ligands point in the opposite direction. Each of these two mol­ecules shows the same coordination as described above, and overall, an *AB*-stacking of the mol­ecules along [001], similar to the hexa­gonal closest packing, is obtained. The crystal structure is shown in Fig. 3 ▸. It should be noted that no acceptor atom for the hydrogen atom H2*A* is present in the neighbourhood within the range of the sum of the van der Waals radii of H and N atoms. Numerical details of the hydrogen bonding are given in Table 1 ▸.

## Synthesis and crystallization   

400 mg (2.41 mmol) of AgSCN were placed in a flame-dried Schlenk tube under argon. Approximately 0.5 ml of liquid ammonia were condensed into the reaction vessel. The reaction vessel was stored at 237 K. After two weeks, colorless crystals of suitable size for X-ray diffraction were obtained from the colorless solution. The formation of the title compound is shown in the scheme.

## Refinement   

Crystal data, data collection and structure refinement details are summarized in Table 2 ▸. All hydrogen atoms of the ammine ligands were located from a difference Fourier map and were refined isotropically without further restraints.

## Supplementary Material

Crystal structure: contains datablock(s) I. DOI: 10.1107/S2056989016008823/wm5294sup1.cif

Structure factors: contains datablock(s) I. DOI: 10.1107/S2056989016008823/wm5294Isup2.hkl

CCDC reference: 1482850

Additional supporting information: crystallographic information; 3D view; checkCIF report

## Figures and Tables

**Table 1:** Hydrogen-bond geometry (Å, °)

*D*—H⋯*A*	*D*—H	H⋯*A*	*D*⋯*A*	*D*—H⋯*A*
N2—H2*C*⋯N1^i^	0.89 (4)	2.34 (4)	3.230 (3)	171 (3)
N2—H2*B*⋯N1^ii^	0.83 (5)	2.43 (5)	3.255 (3)	170 (4)
N3—H3*C*⋯N1^iii^	0.90 (4)	2.31 (4)	3.128 (3)	151 (3)
N3—H3*B*⋯N1^iv^	0.83 (4)	2.39 (4)	3.208 (3)	168 (4)
N3—H3*A*⋯S1^ii^	0.87 (4)	2.82 (4)	3.672 (2)	166 (3)

Symmetry codes: (i) 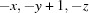; (ii) 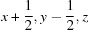; (iii) 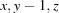; (iv) 
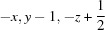
.

**Table 2:** Experimental details

Crystal data	
Chemical formula	[Ag_2_(SCN)_2_(NH_3_)_4_]
*M*_r_	400.04
Crystal system, space group	Monoclinic, *C*2/*c*
Temperature (K)	100
*a*, *b*, *c* (Å)	12.8263 (9), 7.1879 (3), 12.2478 (9)
β (°)	98.936 (6)
*V* (Å^3^)	1115.47 (12)
*Z*	4
Radiation type	Mo *K*α
μ (mm^−1^)	3.85
Crystal size (mm)	0.26 × 0.16 × 0.14

Data collection	
Diffractometer	Stoe *IPDS* 2T
Absorption correction	Numerical (*X-RED32* and *X-SHAPE*; Stoe & Cie, 2009 ▸)
*T*_min_, *T*_max_	0.768, 0.918
No. of measured, independent and observed [*I* > 2σ(*I*)] reflections	7257, 1690, 1593
*R*_int_	0.028
(sin θ/λ)_max_ (Å^−1^)	0.714

Refinement	
*R*[*F* ^2^ > 2σ(*F* ^2^)], *wR*(*F* ^2^), *S*	0.029, 0.061, 1.11
No. of reflections	1690
No. of parameters	79
H-atom treatment	All H-atom parameters refined
Δρ_max_, Δρ_min_ (e Å^−3^)	1.26, −1.82

Computer programs: *X-AREA* (Stoe & Cie, 20011 ▸), *X-RED32* (Stoe & Cie, 2009 ▸), *SHELXT* (Sheldrick, 2015*a* ▸), *SHELXL2014* (Sheldrick, 2015*b* ▸), *SHELXLE* (Hübschle *et al.*, 2011 ▸), *DIAMOND* (Brandenburg, 2015 ▸) and *publCIF* (Westrip, 2010 ▸).

## supplementary crystallographic information

### Crystal data

**Table:**

[Ag_2_(SCN)_2_(NH_3_)_4_]	*F*(000) = 768
*M**_r_* = 400.04	*D*_x_ = 2.382 Mg m^−^^3^
Monoclinic, *C*2/*c*	Mo *K*α radiation, λ = 0.71073 Å
*a* = 12.8263 (9) Å	Cell parameters from 13119 reflections
*b* = 7.1879 (3) Å	θ = 3.2–35.2°
*c* = 12.2478 (9) Å	µ = 3.85 mm^−^^1^
β = 98.936 (6)°	*T* = 100 K
*V* = 1115.47 (12) Å^3^	Block, colorless
*Z* = 4	0.26 × 0.16 × 0.14 mm

### Data collection

**Table:**

Stoe IPDS 2T diffractometer	1690 independent reflections
Radiation source: sealed X-ray tube, 12 x 0.4 mm long-fine focus	1593 reflections with *I* > 2σ(*I*)
Plane graphite monochromator	*R*_int_ = 0.028
Detector resolution: 6.67 pixels mm^-1^	θ_max_ = 30.5°, θ_min_ = 3.2°
rotation method scans	*h* = −18→18
Absorption correction: numerical (*X-RED32* and *X-SHAPE*; Stoe & Cie, 2009)	*k* = −10→9
*T*_min_ = 0.768, *T*_max_ = 0.918	*l* = −17→17
7257 measured reflections	

### Refinement

**Table:**

Refinement on *F*^2^	0 restraints
Least-squares matrix: full	Hydrogen site location: difference Fourier map
*R*[*F*^2^ > 2σ(*F*^2^)] = 0.029	All H-atom parameters refined
*wR*(*F*^2^) = 0.061	*w* = 1/[σ^2^(*F*_o_^2^) + (0.0131*P*)^2^ + 6.0057*P*] where *P* = (*F*_o_^2^ + 2*F*_c_^2^)/3
*S* = 1.11	(Δ/σ)_max_ < 0.001
1690 reflections	Δρ_max_ = 1.26 e Å^−^^3^
79 parameters	Δρ_min_ = −1.82 e Å^−^^3^

### Special details

**Table:**

Geometry. All esds (except the esd in the dihedral angle between two l.s. planes) are estimated using the full covariance matrix. The cell esds are taken into account individually in the estimation of esds in distances, angles and torsion angles; correlations between esds in cell parameters are only used when they are defined by crystal symmetry. An approximate (isotropic) treatment of cell esds is used for estimating esds involving l.s. planes.

### Fractional atomic coordinates and isotropic or equivalent isotropic displacement parameters (Å^2^)

**Table:**

	*x*	*y*	*z*	*U*_iso_*/*U*_eq_	
Ag1	0.06012 (2)	0.01303 (3)	0.14990 (3)	0.04102 (10)	
S1	−0.13234 (4)	0.10809 (8)	0.10715 (5)	0.01844 (11)	
N1	−0.11243 (17)	0.4982 (3)	0.10139 (18)	0.0226 (4)	
N2	0.17384 (18)	0.2371 (3)	0.11205 (19)	0.0217 (4)	
N3	0.10144 (16)	−0.2907 (3)	0.16630 (18)	0.0189 (4)	
C1	−0.11906 (16)	0.3371 (3)	0.10457 (17)	0.0162 (4)	
H2A	0.187 (3)	0.315 (6)	0.169 (3)	0.037 (10)*	
H2B	0.228 (3)	0.181 (7)	0.101 (4)	0.050 (12)*	
H2C	0.150 (3)	0.311 (5)	0.055 (3)	0.034 (9)*	
H3A	0.158 (3)	−0.322 (5)	0.140 (3)	0.029 (9)*	
H3B	0.110 (3)	−0.331 (5)	0.231 (3)	0.033 (9)*	
H3C	0.050 (3)	−0.355 (6)	0.125 (3)	0.039 (10)*	

### Atomic displacement parameters (Å^2^)

**Table:**

	*U*^11^	*U*^22^	*U*^33^	*U*^12^	*U*^13^	*U*^23^
Ag1	0.02898 (12)	0.02005 (11)	0.0765 (2)	0.00256 (8)	0.01589 (11)	0.01630 (11)
S1	0.0185 (2)	0.0165 (2)	0.0201 (2)	−0.00265 (19)	0.00216 (18)	0.00010 (19)
N1	0.0216 (9)	0.0201 (10)	0.0247 (10)	0.0003 (7)	−0.0008 (7)	−0.0002 (8)
N2	0.0245 (10)	0.0190 (9)	0.0220 (10)	0.0006 (8)	0.0048 (8)	0.0022 (8)
N3	0.0177 (9)	0.0204 (9)	0.0191 (9)	0.0000 (7)	0.0045 (7)	0.0008 (7)
C1	0.0139 (9)	0.0217 (10)	0.0126 (8)	0.0000 (7)	0.0009 (7)	−0.0011 (8)

### Geometric parameters (Å, º)

**Table:**

Ag1—N3	2.248 (2)	N2—H2A	0.89 (4)
Ag1—N2	2.269 (2)	N2—H2B	0.83 (5)
Ag1—S1	2.5363 (6)	N2—H2C	0.89 (4)
Ag1—S1^i^	3.0533 (7)	N3—H3A	0.87 (4)
Ag1—Ag1^i^	3.0927 (6)	N3—H3B	0.83 (4)
S1—C1	1.656 (2)	N3—H3C	0.90 (4)
N1—C1	1.162 (3)		
			
N3—Ag1—N2	123.89 (8)	Ag1—N2—H2C	115 (2)
N3—Ag1—S1	119.24 (6)	H2A—N2—H2C	104 (3)
N2—Ag1—S1	113.74 (6)	H2B—N2—H2C	111 (4)
N3—Ag1—Ag1^i^	93.95 (5)	Ag1—N3—H3A	115 (2)
N2—Ag1—Ag1^i^	125.28 (6)	Ag1—N3—H3B	115 (3)
S1—Ag1—Ag1^i^	64.820 (16)	H3A—N3—H3B	105 (3)
C1—S1—Ag1	99.91 (8)	Ag1—N3—H3C	108 (3)
Ag1—N2—H2A	109 (3)	H3A—N3—H3C	104 (3)
Ag1—N2—H2B	105 (3)	H3B—N3—H3C	109 (3)
H2A—N2—H2B	113 (4)	N1—C1—S1	178.2 (2)

Symmetry code: (i) −*x*, *y*, −*z*+1/2.

### Hydrogen-bond geometry (Å, º)

**Table:**

*D*—H···*A*	*D*—H	H···*A*	*D*···*A*	*D*—H···*A*
N2—H2*C*···N1^ii^	0.89 (4)	2.34 (4)	3.230 (3)	171 (3)
N2—H2*B*···N1^iii^	0.83 (5)	2.43 (5)	3.255 (3)	170 (4)
N3—H3*C*···N1^iv^	0.90 (4)	2.31 (4)	3.128 (3)	151 (3)
N3—H3*B*···N1^v^	0.83 (4)	2.39 (4)	3.208 (3)	168 (4)
N3—H3*A*···S1^iii^	0.87 (4)	2.82 (4)	3.672 (2)	166 (3)

Symmetry codes: (ii) −*x*, −*y*+1, −*z*; (iii) *x*+1/2, *y*−1/2, *z*; (iv) *x*, *y*−1, *z*; (v) −*x*, *y*−1, −*z*+1/2.
